# Genotypic but not phenotypic historical contingency revealed by viral experimental evolution

**DOI:** 10.1186/1471-2148-13-46

**Published:** 2013-02-19

**Authors:** Stéphanie Bedhomme, Guillaume Lafforgue, Santiago F Elena

**Affiliations:** 1 Instituto de Biología Molecular y Celular de Plantas, Consejo Superior de Investigaciones Científicas-UPV, 46022, València, Spain; 2 The Santa Fe Institute, Santa Fe, 87501, New Mexico; 3 Present address: Infections and Cancer, Catalan Institute of Oncology (ICO), Barcelona, Spain

## Abstract

**Background:**

The importance of historical contingency in determining the potential of viral populations to evolve has been largely unappreciated. Identifying the constraints imposed by past adaptations is, however, of importance for understanding many questions in evolutionary biology, such as the evolution of host usage dynamics by multi-host viruses or the emergence of escape mutants that persist in the absence of antiviral treatments. To address this issue, we undertook an experimental approach in which sixty lineages of *Tobacco etch potyvirus* that differ in their past evolutionary history and degree of adaptation to *Nicotiana tabacum* were allowed to adapt to this host for 15 rounds of within host multiplication and transfer. We thereafter evaluated the degree of adaptation to the new host as well as to the original ones and characterized the consensus sequence of each lineage.

**Results:**

We found that past evolutionary history did not determine the phenotypic outcome of this common host evolution phase, and that the signal of local adaptation to past hosts had largely disappeared. By contrast, evolutionary history left footprints at the genotypic level, since the majority of host-specific mutations present at the beginning of this experiment were retained in the end-point populations and may have affected which new mutations were consequently fixed. This resulted in further divergence between the sequences despite a shared selective environment.

**Conclusions:**

The present experiment reinforces the idea that the answer to the question “How important is historical contingency in evolution?” strongly depends on the level of integration of the traits studied. A strong historical contingency was found for TEV genotype, whereas a weak effect of on phenotypic evolution was revealed. In an applied context, our results imply that viruses are not easily trapped into suboptimal phenotypes and that (re)emergence is not evolutionarily constrained.

## Background

One of the main goals of evolutionary biology is to understand the process leading to the observed patterns of phenotypic diversity. Natural selection, historical events and chance have been identified as factors shaping diversity at different scales, from local adaptation to speciation [1,2]. These evolutionary processes are not mutually exclusive and often contribute together to the pattern of differentiation. While natural selection leads to a deterministic adaptation to environmental conditions, historical factors and chance can produce different outcomes despite similar environmental conditions. The idea of contingency playing a role in the evolution and generation of biological diversity was actually central in Darwin’s work and a key point differentiating his theory from the ones of his contemporaries. Chance plays a role both in the initial generation of diversity, *i.e.* mutation, and in the maintenance or elimination of the diversity in the population, *i.e.* genetic drift. History might play a role if initial differences in the phenotype and/or the genotype affect adaptation.

In this context, as outlined by Travisano *et al.*[1] and Blount *et al.*[3], we will define historical contingency as an evolutionary situation where the initial phenotype and/or genotype influences the response to a selection episode and the evolutionary outcome in terms of phenotype and/or genotype. There are various approaches for studying the importance of historical contingency, the choice of which strongly depends on the possibility of performing experimental evolution. First, for large complex organisms, a number of studies have characterized groups (from population to species) adapting in parallel to similar environments, in the context of adaptive radiation. In this class of studies, related species (*e.g.*, anole lizards [4,5], mosquitofishes [6], or orb-weaving spiders [7]) or populations (*e.g.*, of mosquitofish [8] or of a freshwater isopod [9]) are characterized at the morphological or behavioral level. The differences found are attributed to the difference in selection pressures, the replicate population and the interaction between these two factors. Some studies control for the phylogeny, so that they can distinguish between the influence of past evolutionary events and specificities of each replicate in determining the current pattern of differentiation. The broad picture coming out of these studies is that the environment is the first determinant of the phenotype but historical events usually also have significant effects, although of lower magnitude.

For organisms amenable to experimental evolution, replaying part of an evolution experiment can also test the importance of historical contingency. This is possible by building “fossil records” along the experimental evolution, as in the case of viruses [10] or bacteria [3,11]. In this last study, by generation 31,500 of the long-term experiment evolution [12], one of the 12 lines of *E. coli* B became able to metabolize citrate. Blount *et al.*[3] determined that the probability of this evolutionary event is much higher when experimental evolution is replayed from samples frozen shortly before its initial appearance. This result highlights the importance of historical contingency and favors the hypothesis that new traits emerge by the occurrence of a series of mutations in a specific order rather than being the result of a unique rare mutation [3,11].

An alternative version of the “replay experiment” is to evolve in a common environment lines or populations that initially differ for known phenotypic and/or genotypic characteristics. The initial diversity can be generated by a strong founder effect in complex organism such as anole lizards [13], a previous phase of experimental evolution (*e.g.*, [1,14,15]) or be present in a mutant or isolate collection (*e.g.*, [16-19]). A variation of this general protocol is to start the experimental evolution with identical populations, place them in a similar environment but vary the conditions of adaptation (*e.g.*, [20]), the size of the transfer bottleneck or the presence of a mutagen. The lesson learned from these studies is that the influence of historical contingencies on evolution strongly depends on the trait measured. Historical contingency tends to have a lower impact on traits that determine fitness than on those that have weak impact on fitness [1,14]. Moreover, a form of historical contingency is systematically found in studies that analyze DNA sequences [15,16,20].

If past historical events constitute evolutionary constraint, identifying its impact on viral evolution is of great importance for understanding the evolution of host-usage dynamics by multi-host viruses or the emergence of escape mutants that persist in the absence of antiviral treatments. Among the few studies addressing the question of historical contingency in virus evolution, Burch and Chao identified two populations of different evolvability during fitness recovery after a mutation accumulation experiment in the bacteriophage ϕ6 [10]: one was climbing a fitness peak whereas the other was at the top of a second, less optimal, fitness peak [21]. In a second study, Herrera *et al.* explored the role of contingency in the coevolutionary process between cells and *Foot-and-mouth disease virus* during persistent infections [22]. Independently evolved lineages that started with the same original viral and cell clones, fixed the same mutations and showed a strong role for historical contingency: the presence of a given pair of mutations in early stages of the coevolutionary process determined the subsequent fixation of other mutations. Finally, in the *Rice yellow mottle virus* (RYMV), it has been demonstrated that the different resistance-breaking mutations of isolates from different cultivars or species cannot be explained by a classical arms race between host and pathogen but result from epistasis between a previously polymorphic site and the site conferring the resistance breaking phenotype [23].

In the present study, we used populations of *Tobacco etch virus* (TEV) generated by Bedhomme *et al.*[24] to assess the importance of historical contingency in the evolution of this virus. The initial experiment was designed to analyze the adaptation of TEV to different host species and to contrast generalist and specialist strategies in the context of adaptation to new hosts [24]. Starting from a single infectious clone, we derived two types of evolutionary histories: (1) viral populations transmitted on the same host; and (2) viral populations transmitted on alternate hosts [24]. The first case was expected to select for specialists whereas the second should have favored generalists. The phenotypic characterization of the evolved lineages allowed us to identify a pattern of higher infectivity and virulence on host(s) present during experimental evolution, which indicates the existence of local adaptation in the majority of the host × evolutionary history combinations [24]. Local adaptation comes, in some of the cases, at a cost on alternative hosts. We did not find any specific characteristics for the alternate-host infecting lineages. Moreover, we did not find a cost for being a generalist. The full-genome consensus sequences of the evolved lineages revealed the fixation of some host-specific mutations but a low level of parallel evolution [24]. These independently evolved lineages, characterized for some phenotypic traits and for their full-genome consensus sequence, constituted an ideal material to investigate historical contingency: we have been able to ask if their initial characteristics affected their phenotype and genotype after a new phase of evolution in a common host. Moreover, the common host chosen for this second phase of evolution was *Nicotiana tabacum*, to which the ancestral infectious clone is presumably adapted [25]. Consequently, evolving all the differentiated lineages on *N. tabacum* constitutes a reverse evolution experiment.

TEV genome is characterized by pervasive epistasis and in particular by a high frequency of reciprocal sign epistasis [26]. This is predicted to produce a highly rugged adaptive landscape, in which many adaptive pathways are inaccessible [27,28]. Moreover, it is known that the sign and the magnitude of epistasis between mutations vary from one host to another for TEV [29]. Such epistasis suggests an important role of historical contingency in TEV, at least at the genotypic level. We made the following predictions: (1) if historical contingency plays a role in phenotypic evolution, the phenotypes at the end of the “common environment” phase will not be the same for all lineages and will depend on the phenotypes at the beginning of this phase and (2) if historical contingency plays a role in genotypic evolution, the level of sequence convergence will be low and the genetic difference between lineages that differ in evolutionary history before the “common-environment” phase will be higher than between the lineages of identical evolutionary history. In terms of adaptive landscape, a significant historical contingency would imply that *N. tabacum* represents an environment with multiple fitness peaks, whereas a lack of historical contingency would suggest that the ancestral host represents an environment with a single accessible peak [30].

## Results

The current experiment was started with 60 lineages grouped in six evolutionary histories. Three evolutionary histories consisted of 15 serial transfers on a same host while the other three used alternate hosts (Additional file 1: Figure S1). These 60 lineages had been characterized for their infectivity and their virulence on the four host plant species used in experimental evolution Phase 1 and their full genome sequence had been obtained. To evaluate the impact of historical contingencies on virus evolution, these 60 lineages were further evolved for 15 passages on a common host, *N. tabacum*. At the end of this second phase of experimental evolution (Phase 2), the infectivity and two virulence index of the 60 lineages were measured for a subset of “evolutionary history” × “host” plant combinations. The full genome consensus sequence was also obtained.

### Infectivity and virulence at the end of phase 1 do not allow predicting infectivity and virulence after phase 2

The main goal of this study was to evaluate the impact of historical contingencies during Phase 2. A first way to look at this is to ask whether the characteristics of the different lineages measured at the end of Phase 1 significantly explain the characteristics of the lineages at the end of Phase 2. This is possible because traits have been measured exactly the same way at the end of the two phases and with a similar level of replication (three plants per lineage per host species combination at the end of Phase 1). The unit of analysis was the lineage. Because infectivity data do not meet the hypothesis of normal distribution, we calculated a Spearman’s rank correlation coefficient. A separate analysis was performed for each host, as it is established that the host effect is very strong. The rank correlation between infectivities at the end of Phase 1 and Phase 2 was not significant for measures on *N. benthamiana* (ρ = 0.005, 28 d.f., *P* = 0.729), *N. tabacum* (ρ < 0.001, 60 d.f., *P* = 0.925) and *C. annuum* (ρ = 0.002, 40 d.f., *P* = 0.809). The rank correlation was significant and positive for infectivity measured on *D. stramonium* (ρ = 0.208, 31 d.f., *P* = 0.008). For virulence data, an ANCOVA was performed for each of the two virulence indices (*V*_*W*_ and *V*_*S*_) using host, virulence index at the end of Phase 1 (as a covariate) and their interaction as factors and the same virulence index at the end of Phase 2 as variable. The ANCOVAs revealed that host had a significant effect for both virulence indices at the end of Phase 2. However, neither virulence indices at the end of Phase 1, nor its interaction with host, had a significant effect on the virulence index at the end of Phase 2 (Table 1).

**Table 1:** ANCOVA with host, virulence index in Phase 1 (host specialization phase) and their interaction as factors and the corresponding virulence index in Phase 2 (common host phase) as variable

**Factors**	**d.f.**	***F***	***P***
*Virulence expressed on size*			
Host	3	43.770	<0.001
Virulence Phase 1	1	0.206	0.651
Host × Virulence Phase 1	3	0.651	0.584
*Virulence expressed on weight*			
Host	3	11.294	<0.001
Virulence Phase 1	1	1.599	0.209
Host × Virulence Phase 1	3	1.298	0.279

### The signature of host specialization detected at the end of phase 1 disappeared after the common host phase

**Figure 1:**
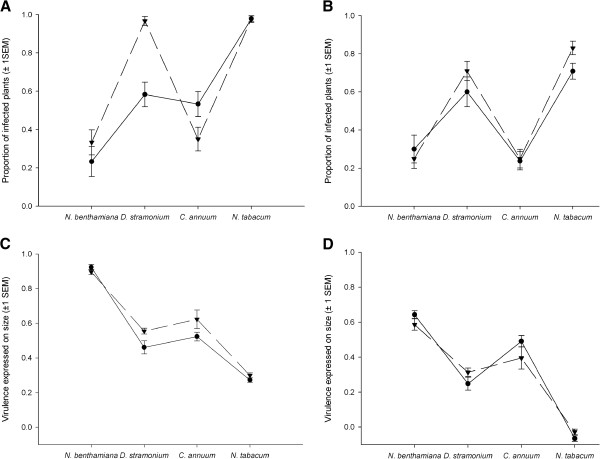
**Infectivity and virulence expressed on size.** Infectivity (**A** and **B**) and virulence expressed on size (**C** and **D**) for local and foreign inoculations after the host specialization phase (**A** and **C**) and after the common host phase (**B** and **D**). Circles and continuous lines represent foreign inoculations. Local inoculations are represented by triangles and dashed lines. All values are represented ±1 SEM.

A second way to look at the impact of historical contingencies on evolution is to analyze if the pattern of local adaptation observed at the end of Phase 1 persisted at the end of Phase 2. This pattern was particularly clear for the variables infectivity and *V*_*S*_*.* The data set of Phase 1 was restricted to “evolutionary history” × “host” combinations used in the phenotypic measurement at the end of Phase 2. For both phases, each inoculation was classified as foreign (lineage inoculated on a host absent for this evolutionary history during Phase 1) or local (lineage inoculated on a host present for this evolutionary history during Phase 1). For each phase, a nominal logistic regression using “host”, “foreign/local” and their interaction as factors and infectivity as variable was performed. The very significant effect of the “host” × “foreign/local” at the end of Phase 1, owing to the strong signal of local adaptation on *D. stramonium,* was absent at the end of Phase 2 (Figure 1A and B and Table 2). ANOVAs using “host”, “foreign/local” and their interaction as factors and *V*_*S*_ or *V*_*W*_ as variable were then run on Phase 1 and Phase 2 data sets. The significant effect of the “foreign/local” factor on *V*_*S*_ at the end of Phase 1 was due to a higher virulence of the local lineages on *D. stramonium* and *C. annuum*. This pattern vanished during Phase 2 (Figure 1C and D and Table 3). *V*_*W*_ had no significant effect of the “foreign/local” factor or of the interaction neither at the end of Phase 1 nor at the end of Phase 2 (Table 3). Therefore, we can conclude that previous phenotypic differences among lineages did not explain the phenotypic characteristic of the lineages after 15 passages into a common host.

**Table 2:** Local adaptation signal expressed on infectivity at the end of the host specialization phase (Phase 1) and at the end of the common host phase (Phase 2)

**Factor**	**d.f.**	χ^**2**^	***P***
*Host specialization phase*			
Host	3	194.03	<0.001
Foreign/local	1	3.62	0.057
Host × foreign/local	3	30.45	<0.001
*Common host phase*			
Host	3	147.273	<0.001
Foreign/local	1	1.683	0.195
Host × foreign/local	3	3.713	0.294

**Table 3:** Local adaptation signal expressed on virulence at the end of the host specialization phase (Phase 1) and at the end of the common host phase (Phase 2)

**Factor**	**d.f.**	***F***	***P***
*Virulence expressed on size – host specialization phase*			
Host	3	167.348	<0.001
Foreign/local	1	5.245	0.023
Host × foreign/local	3	1.939	0.123
*Virulence expressed on size – common host phase*			
Host	3	211.975	<0.001
Foreign/local	1	0.279	0.598
Host × foreign/local	3	2.660	0.048
*Virulence expressed on weight* – *host specialization phase*			
Host	3	14.569	<0.001
Foreign/local	1	0.627	0.429
Host × foreign/local	3	1.161	0.325
*Virulence expressed on weight* – *common host phase*			
Host	3	25.840	<0.001
Foreign/local	1	0.755	0.386
Host × foreign/local	3	1.791	0.149

### Selection in a variable environment does not improve adaptability

Generalists have sometimes been predicted to have a higher potential for adaptation, raising the interesting question of whether the lineages that alternate between two hosts during Phase 1 show a particular behavior in the adaptation to a common host. To answer this question, we used a data set containing only the phenotypic data measured on *N. tabacum*. A nominal logistic regression was performed with “generalist/specialist”, “evolutionary history” nested within “generalist/specialist” and “replicate evolutionary history” nested within “evolutionary history” as factors and infectivity as variable. The “generalist/specialist” factor had no significant effect on infectivity data (χ^2^ < 0.001, 1 d.f., *P* = 0.989). An ANOVA with the same factors was used to analyze the two virulence indices and the “generalist/specialist” factor did not have any significant effect neither on *V*_*S*_ (*F*_1,4_ = 0.650, *P* = 0.422) nor on *V*_*W*_ (*F*_1,4_ = 0.334, *P* = 0.565). Hence, we conclude that generalist and specialist lineages did not differ in their potential to adapt to a new host.

### A large proportion of mutations acquired during phase 1 were conserved in phase 2

**Figure 2:**
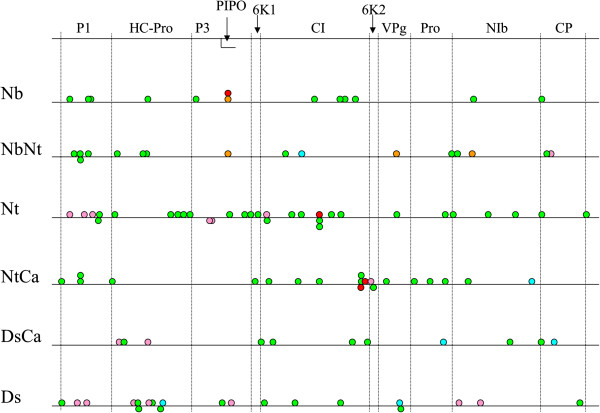
**Schematic representation of the collection of mutations obtained in the 60 experimentally evolved lineages.** The first line represents the full TEV genome with the position of the eleven mature viral proteins within the ORF. The six other lines correspond each to one evolutionary history in Phase 1 and all the mutations obtained for this evolutionary history are represented. In green, new mutations; in blue mutations that got fixed (*i.e.* polymorphic at the end of Phase 1 and fixed for the mutant allele at the end of Phase 2); in red, reversion to the ancestral genotype; in orange, reverting sites (fixed for a mutant allele at the end of Phase 1 and polymorphic mutant/ancestral at the end of Phase 2) and in pink reversions from a polymorphic state (polymorphic mutant/ancestral at the end of Phase 1 and reverted to the ancestral state at the end of Phase 2). Circles piled above the line represent mutations at the exact same locus whereas when several mutations were close in the sequence, they are represented for clarity below the line.

**Figure 3:**
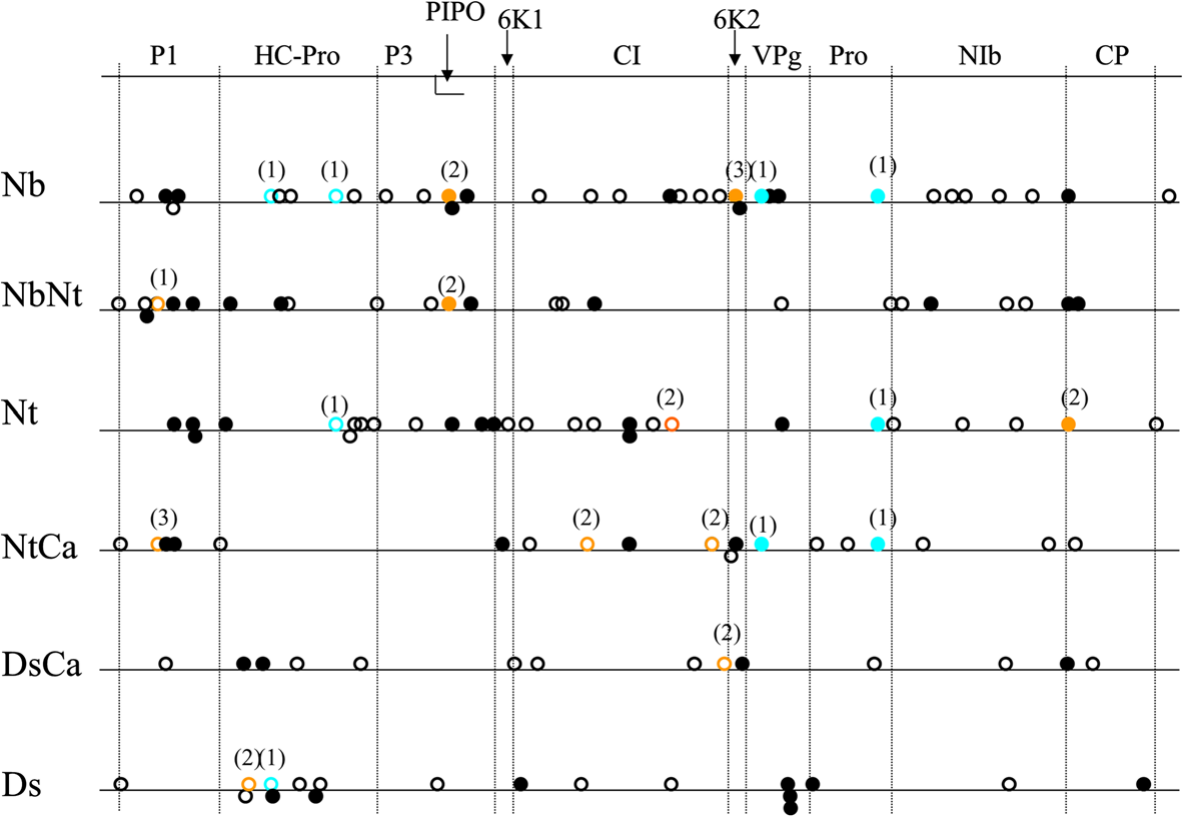
**Snapshot of the collection of mutations obtained at the end of the common host phase.** The first line represents the full TEV genome with the position of the eleven mature viral proteins within the ORF. The six other lines correspond each to one evolutionary history in Phase 1 and all the mutations present at the end of Phase 2 for this evolutionary history are represented. Full circles represent non-synonymous mutations; empty circles represent synonymous mutations. Mutations that are unique in the data set are represented in black, mutations shared at the end of Phase 2 between lineages sharing a same host in Phase 1 are represented in orange and mutations shared at the end of Phase 2 between lineages that do not share a same host in Phase 1 are represented in blue.

During Phase 2, a total of 113 independent mutations occurred at 107 different loci. There were between zero and six mutations per lineage in the 60 independently evolved lineages (see Figure 2 for a graphical representation and Additional file 1: Table S2 for a complete list of mutations). The transition:transversion ratio was six. Sixty-five mutations were synonymous and 46 non-synonymous. Out of the 113 mutations observed, 10 (9%) were not unique. If we look at the sequences obtained at the end of Phase 2 and compare them to their common ancestral sequence, *i.e.* if we take into account the mutations occurred during Phases 1 and 2, there are a total of 150 independent mutations and 131 polymorphic sites (Figure 3). Out of the 150 mutations observed, 32 (21%) were not unique and out of the 131 polymorphic loci identified, 13 (10%) were affected in multiple independent lineages. Interestingly, while at the end of Phase 1, the very large majority (nine out of ten) of the multiply affected sites were found in lineages sharing a host, at the end of Phase 2, four cases of shared mutations were present among lineages that had no host in common during Phase 1 (Figure 3). Finally, a large proportion of the mutations fixed in Phase 1 were conserved: 68 of the 94 mutations detected at the end of Phase 1 were still present, in a fixed or polymorphic state at the end of Phase 2. Having all the end-point sequences available, it is tempting to ask whether a phylogenetic reconstruction accurately recovers the true evolutionary history, as has been done in a previous study by Bull *et al.*[31] which experimental design shared common points with ours (viral adaptation on two hosts and with host switch). Unfortunately, there is not enough signal in our data to reconstruct a well-supported phylogeny: they present from zero to nine point mutations (average 2.57) compared to the ancestral sequence, whereas the endpoint sequences in Bull *et al.*[31] presented from 12 to 33 differences (average 18.11).

### Genetic distances between evolutionary histories increased despite common host selection

**Figure 4:**
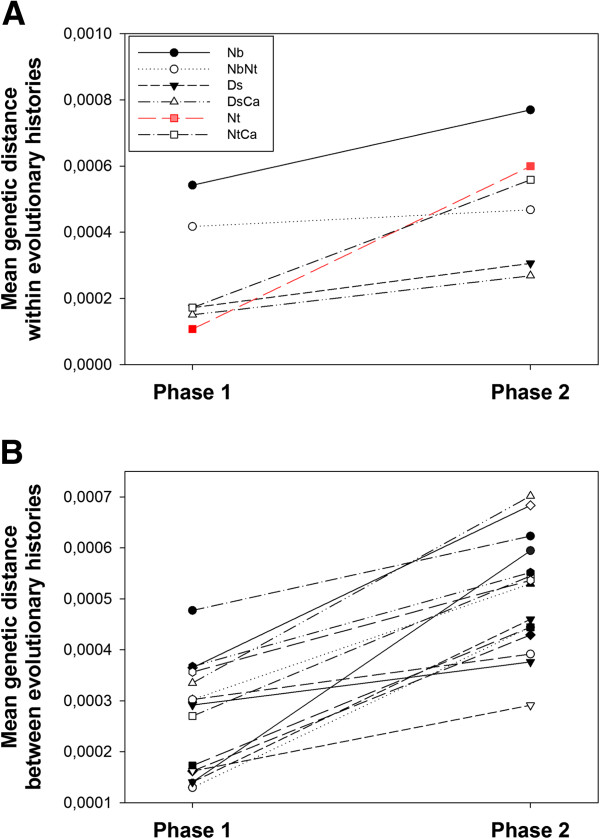
**Changes in mean genetic diversities.** (**A**) Change in mean genetic diversity within evolutionary histories from Phase 1 to Phase 2. Each evolutionary history is indicated by a different symbol and line (inset). In red, the Nt treatment is the only one showing a significant increase. (**B**) Change in mean genetic diversity between evolutionary histories from Phase 1 to Phase 2. Each comparison between pairs of histories is indicated by a different symbol.

The sequences obtained at the end of Phases 1 and 2 were aligned separately and the within groups and net between group mean distances were calculated for each alignment using MEGA version 5 [32], with groups defined as evolutionary histories. Within and between groups genetic distances were computed using the composite maximum likelihood estimator and assuming a uniform rate of substitution among all sites in the genome. Standard errors were estimated by the bootstrap method based on 1000 pseudo-replicates. The average genetic distance within evolutionary histories only increased significantly for the Nt treatment (Figure 4A; *z*-test *P* = 0.008). However, treating each evolutionary history as an independent observation, a paired-samples *t*-test supported a significant overall 89.92% increase in genetic distances within evolutionary histories (average change = 2.345±0.699 ×10^−4^; *P* = 0.020). On the other side, we found that the average genetic distance between evolutionary histories is 91.32% larger at the end of the common host phase than at the end of the host specialization phase (average change = 2.420±0.274 ×10^−4^; paired *t*-test, *P* < 0.001) (Figure 4B). This 3.18% larger average change in genetic differences between evolutionary histories relative to the increase within evolutionary histories further supports the existence of historical contingencies at the genotypic level.

### Genetic diversity at the end of phase 2 can be predicted by the genetic diversity at the end of phase 1

**Figure 5:**
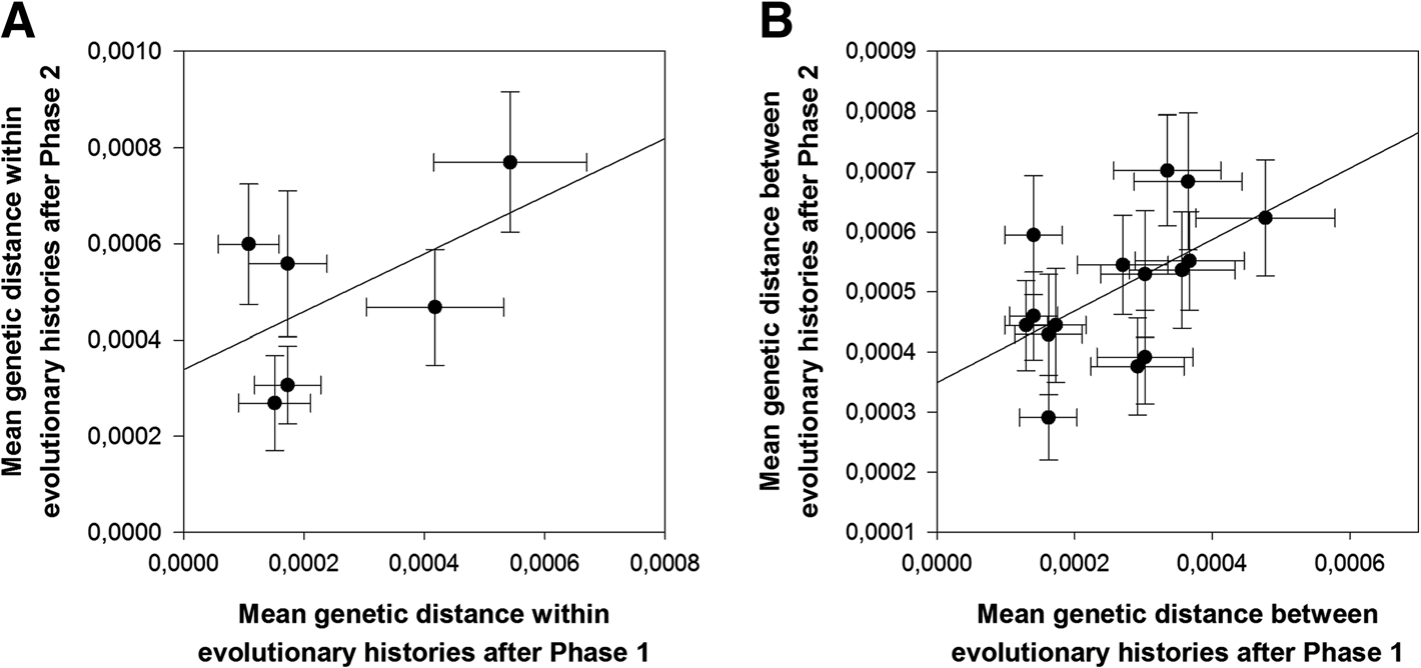
**Relationship between genetic distances after Phases 1 and 2.** (**A**) Observed relationship for genetic distances evaluated within evolutionary histories. (**B**) Observed relationship for genetic distances evaluated between all pairwise evolutionary histories. In both cases error bars represent ±1 SE. The solid line represents the least-squares linear regression.

According to the historical contingency hypothesis, the fingerprint of genetic diversity between evolutionary histories observed at the end of Phase 1 may still be detectable after Phase 2. By contrast, the genetic diversity within histories at the end of the host specialization phase would not necessarily be retained after evolution in the common host *N. tabacum*. To test these two predictions we performed regression analyses at within- and between-treatment diversity across the two evolutionary phases (Figure 5). The regression was not significant at the within evolutionary histories level (Figure 5A; *R*^2^ = 0.314, *F*_1,4_ = 1.833, *P* = 0.247). By contrast, as predicted by the historical contingency hypothesis, the regression was significant at the between evolutionary histories level (Figure 5B; *R*^2^ = 0.303, *F*_1,13_ = 5.640, *P* = 0.034), thus confirming that the amount of genetic diversity existing between evolutionary histories at the end of Phase 1 was retained after 15 additional passages in the common host. The slope of the regression line, 0.594±0.250, was smaller than one, indicating that the dependence of the genetic differences between evolutionary histories in Phase 2 was less important for highly different histories than for similar histories (Figure 5B).

## Discussion

### A low influence of historical contingency at the phenotypic level

At the phenotypic level, the clear pattern of local adaptation that was detected for different traits on different host plants at the end of the “host specialization” [24] phase faded out during the “common host phase”. Moreover, the phenotypic characteristics at the end of Phase 1 do not allow predicting the characteristics at the end of the Phase 2, except in one case (infectivity in *D. stramonium*) meaning that a large part of the differentiation between lineages and evolutionary histories has disappeared and that the lineage collection is now homogeneous in terms of infectivity and virulence. Altogether, the analysis of phenotypic traits revealed the absence or the low influence of historical contingency during the “common-environment phase”. This low influence of historical contingency suggests that the original host *N. tabacum* represents a case of a single accessible fitness peak, or a very limited number of accessible peaks with similar height, otherwise new mutations fixed during Phase 1 might make alternative and more diverse peaks available [30]. There is one exception to this general result: the lineages evolved in the first phase on *C. annuum* that were unable to produce a systemic infection on *N. tabacum* either because they did not infect at all or because they only produced local lesions. For the second category, 15 serial passages of local lesions were performed in a separate experiment; these lineages conserved the ability of producing local lesions but never recovered the ability to generate systemic and symptomatic infections. For these particular lineages, we thus have a case of strong effect of past evolutionary events, as they seem to have ended up in an evolutionary trap without possibility of adaptation to their previous host *N. tabacum*.

The identification of historical contingency is of course dependent on the time scale at which an experiment is performed: the probability of identifying historical contingency is higher if the initial history of differentiation is long or the period of adaptation to a common environment is short. In our case, because we used very similar protocols (in terms of treatment and number of transfers) for the “host specialization” and the “common environment” phases, we know that the experimental evolution was able to generate differences between treatments that were then erased in a second phase, equivalent in time and transfer conditions. The identification of historical contingency also depends on the integration level of the traits measured. We cannot exclude that the evolution of traits of lower integration (*e.g.*, propagation speed of the virus within the plant, effect of the virus on the internode distance) would have a stronger signal of historical contingency, each lineage potentially reaching the same integrated phenotype through alternative evolutionary paths. However, our data show that historical contingency had little influence on the evolution of traits that are classically measured in plant virology because they have an evolutionary and applied meaning. Finally, strongly divergent past histories make more likely the detection of their influence on current evolution. In the present experiment, we chose to use only *Solanacea* as hosts, because TEV produces symptomatic infections in *Solanacea*, which make possible large-scale experimental evolution without molecular detection of infections. We cannot exclude that using more phylogenetically diverse hosts in the “host specialization phase” would have produced phenotypic differences that would not have been erased by the common host environment.

Lineages that had gone through a single-host or an alternate-host evolutionary history during the first phase of experimental evolution do not show significant differences between each other and in particular the “alternate-host” evolutionary history do not show characteristics of a better adaptation to the new common host, *N. tabacum*, as predicted by theoretical models [33,34]. This result, however, is not surprising as no significant difference was identified between the “single-host” and the “alternate-host” at the end of Phase 1.

### Strong historical contingency at the sequence level: a preponderance of compensatory mutations?

At the genotypic level, we see a blurring of the “host-specific mutation” pattern that was present at the end of Phase 1. Moreover, as revealed by the mean genetic distances within and between evolutionary histories at the end of Phase 1 and Phase 2, there is a general increase in distance between the evolutionary histories, which is going in the direction of a strong historical contingency at the sequence level. The genotypic differences between the lineages are not eroded by a phase of “common environment” evolution, but, on the contrary, these differences seem to have been at the origin of additional differences. Indeed, the amount of genetic differences among histories has increased almost twice as much as the amount of genetic differences within histories (Figure 4). The majority of the mutations (80%) that happened during the “common host” phase correspond to new mutations or to fixation of mutations that were present in a polymorphic state at the end of Phase 1, whereas the total or partial reversions to the ancestral sequence represent only 20% of the mutations. Non-reversion mutations are likely of two classes: (1) neutral mutations fixed by chance and (2) compensatory mutations mitigating the negative effects of mutations in *N. tabacum*, fixed during virus replication in other hosts, and responsible for antagonistic pleiotropy. Determining to which of these two categories each mutation belongs would require building infectious clones by directed mutagenesis to know whether each mutation acquired during the second phase provides a fitness advantage in *N. tabacum* in the specific genetic background in which it was fixed (*i.e.*, the sequence at the end of Phase 1). However, it is very unlikely that all the mutations fixed during the second phase are neutral and that the Phase 2 of the experiment boils down to the differentiation of isolated populations by genetic drift. Indeed, we know from Phase 1 that our experimental evolution protocol is able to trigger the appearance of local adaptation and host-specific mutations in the context of evolution on (a) new host(s), *i.e.* that not all changes are stochastic. Phase 2 actually is a phase of adaptation to a new host for the majority of the lineages and based on the phenotypic changes occurring during this phase, we can be sure that at least part of the mutations acquired was not neutral. Finally, it is possible to predict the “among evolutionary history genetic diversity” at the end of Phase 2 using this same diversity index measured at the end of Phase 1 (Figure 5). In the same way as the absence of predictability at the phenotypic level is interpreted as an absence of historical contingency, the significant values at the genotypic level reinforce the idea that historical contingency plays a role for genotypic evolution.

Previous reverse evolution experiments in bacteria and viruses have shown that compensatory mutations are far more frequent than reversion [35-37] (for reviews, see [30,38]). The magnitude of fitness recoveries is larger for reversions than for compensatory mutations; therefore, we could expect reversion to be the preponderant mutation type in reverse evolution experiments. However, in organisms like bacteria and viruses, where sufficient relevant genetic variation is generated by *de novo* mutations, at least two genetic mechanisms impeding reverse evolution at the sequence level have been identified [39]: first, due to genotype × environment interaction, the alleles fixed in another environment are not necessarily detrimental in the ancestral conditions and if neutral, they will not be eliminated during reverse evolution. Second, forward evolution might have fixed favorable combinations of epistatic mutations. Reverse evolution requires breaking-up these combinations. This second mechanism is particularly likely in TEV, owing to the pervasive epistasis detected in its genome [26]. Compensatory mutations have a higher probability to appear because there are several potential compensatory mutations for the same initial mutation whereas there is only one possible reversion. For example, ten different compensatory mutations have been identified for the cost of the *rpsL* mutation conferring resistance to streptomycin in *E. coli*[40]. This higher probability of appearance translates, in particular in the context of passages with narrow bottlenecks, into a higher probability of fixation. Indeed, compensatory mutations will tend to appear earlier within a passage and have time to spread in the population, because of their fitness advantage, before the reversion occurs. The presence, at very high frequency, of a compensatory mutation reduces the fitness advantage, and thus the spreading speed, of the reversion such that the frequency of the reversion is in many cases small when the bottleneck takes place [40]. This last evolutionary phenomenon is very likely to have taken place in our evolution experiment, as the experimental procedure of serial transfers by mechanical inoculation imposed narrow bottlenecks. Transmission of this type, although artificial in our case, is frequent in natural transmission of virus and other pathogens and the interaction between the probability of appearance of compensatory and reversion mutations, clonal interference and bottlenecks are likely to play a substantial role in the low reversibility at the sequence level. All in all, there are many reasons identified for the higher frequency of compensatory evolution than reversion and our study contributes to the growing experimental support to this important determinant of historical contingency at the sequence level.

The opposite conclusion obtained at the genotypic and the phenotypic level highlights that the relationship between genotype and phenotype is far from being a bijective map, even in small genomes like the viral ones. This is a direct consequence of the omnipresence of epistasis and of compensatory evolution.

### Are the Nt populations drifting through neutral networks?

One puzzling result is the high number of new mutations accumulated by the Nt lineages during the second phase of experimental evolution (Figure 2). These lineages are supposed to represent a control as they have been passaged, in both phases of experimental evolution, only on *N. tabacum*, the ancestral host. For this reason, they were expected to accumulate fewer mutations than other evolutionary histories. A potential explanation is that because these lineages are pre-adapted to the host, the majority of the mutations that occur are neutral or slightly deleterious whereas there is a higher probability for beneficial mutations in other hosts [41]. This difference in the distribution of fitness effects of mutations provides more opportunities for selective sweeps in other hosts than in *N. tabacum* and these selective sweeps might have eliminated in other hosts mutations of small effects that fixed in the Nt lineages. This explanation shares strong analogies with the model of neutral networks that was first developed at the theoretical level [42] and then applied to the antigenic evolution of *Influenza A virus*[43,44]. According to this model, evolving viral populations alternate epochs of phenotypic stasis punctuated by sudden changes in the phenotype, selected by the adaptive immunity of the host. However, phenotypic stasis does not necessarily mean genotypic stasis as neutral mutations accumulate allowing the population to drift continuously through this neutral network until jumping to a different one. In our case, the phase of adaptation to a new host would represent an epoch of strong selection and hence rapid phenotypic innovation. Once adapted to this new host, the population enters in a drifting phase in which only neutral mutations accumulate. In the case of Nt lineages, Phase 2 corresponds to such a situation, thus accumulating a large number of neutral mutations. By contrast, in the other lineages, Phase 2 represents a succession of jumps among neutral networks that improve adaptation to the new host *N. tabacum* and these selective sweeps erase population variation.

### Historical contingency and fitness landscape

The concept of historical contingency in evolution is intimately related to the shape of the fitness landscape [45]. Landscape topologies lie between two extremes: smooth and rugged (or uncorrelated) landscapes. Smooth landscapes only possess one fitness peak whereas rugged landscapes possess several ones, separated by fitness valleys. Ruggedness corresponds to a high level of epistasis, which implies that the effect of one mutation depends on the genetic background and consequently, positions close in genotype do not necessarily have similar fitness. At the end of the experiment, the evolved lineages have similar phenotypic characteristics for traits related to fitness. This could represent a case of a unique adaptive peak or a case of various peaks of equivalent fitness, spread over a rugged landscape, and potentially connected between them. The genotypic data, combined to the presence of strong epistatic interactions in this species, allow us to say that the genotypic landscape is necessarily rugged, as the lineages have a variety of genotypes and thus lie in different places of the landscape. However, fully understanding the constraints leading to this particular situation would require characterizing the intermediate steps between the sequences present at the end of the evolution.

## Conclusion

The present experiment, by studying sequence and phenotypic evolution on the same system, reinforces the idea that the answer to the question “How important is historical contingency in evolution?” strongly depends on the level of integration of the traits studied [46]. At the genotypic level, a strong historical contingency was found, likely explained by the preponderance of compensatory evolution over reversion, whereas a weak effect of historical contingencies on the phenotypic evolution was revealed. In the context of host switch and (re)emerging diseases, our results strengthen the idea that viruses are not easily trapped into suboptimal phenotypes. Except in the special case of adaptation to *C. annuum*, TEV is able to readapt rapidly to a previous host whatever its evolutionary history has been. This strong adaptive potential seems to be partially due to the possibility to reach the same fitness through various evolutionary pathways. It means that even if a viral infection is temporarily absent, any other species of the range acts as a reservoir. Reemergence is very likely and its success depends more on ecological conditions than on evolutionary constraints.

## Methods

### Viruses

Our model system is TEV (genus *Potyvirus*, family *Potyviridae*). TEV has a moderately wide host range and most natural hosts belong to the *Solanaceae* family [47]. It has a positive-single-strand RNA genome of 9.5 kb that encodes a large polyprotein, which is auto-catalytically cleaved into ten multifunctional mature viral proteins [48]. Recently, an overlapping ORF coding a small additional protein after frame-shifting has been discovered [49]. A virus-encoded RNA-dependent RNA-polymerase that lacks proofreading activity replicates the viral genome. TEV mutation rate is thus high, estimated to be around 10^−5^ to 10^−6^ mutations per site and per generation [50].

The derivation of the lineages used to start the present study can be found in [24] and in Additional file 1: Figure S1. Briefly, these lineages are the product of an evolution experiment initiated with an infectious clone of TEV [51]. The TEV genome used to generate this clone has been isolated from *N. tabacum*[25] and its sequence is published elsewhere [52]*.* The experimental evolution design contained seven evolutionary histories. In four of them, the viral populations were serially passaged (15 passages by mechanical inoculation) on the same host, denominated as lineages Nb (*Nicotiana benthamiana*), Ds (*Datura stramonium*), Ca (*Capsicum annuum*), and Nt (*N. tabacum*). In the three other evolutionary histories, the viruses were serially passaged on alternate hosts using the following pairs: *N. benthamiana* and *N. tabacum* (NbNt), *N. tabacum* and *C. annuum* (NtCa) and *D. stramonium* and *C. annuum* (DsCa). The four host species used belong to the *Solanacea* family and TEV produces systemic symptoms in all of them. This evolution experiment, which represents a “host specialization phase” will be denominated Phase 1 hereafter. For each evolutionary history, ten independent replicates were performed.

### Experimental evolution on a common host

The goal of this second phase of experimental evolution was to test for the role of historical contingencies when lineages that differed phenotypically and genotypically were put on the same host and consequently under the same selection pressures. The common host was *N. tabacum* (Additional file 1: Figure S1).

Saps of equivalent viral RNA concentrations were prepared by mixing infected tissue from the 15^th^ passage of Phase 1 and inoculation buffer (100 mg mL^-1^ Carborundum, 0.5 M K_2_HPO_4_). For each replicate, two plants of *N. tabacum* were mechanically inoculated with 5 μL of this sap on one leaf. For the subsequent passages, at seven days post-inoculation (dpi), the aerial part of one of the two plants in each lineage was collected. If the two plants were systematically infected (which occurred in more than 95% of the cases), the collected plant was chosen randomly. If only one was systematically infected, this one was collected. In both cases, the inoculated leaf was removed and a sap was prepared with 150 mg of infected tissue in 1 mL of inoculation buffer. For each lineage, two plants were then inoculated on one leaf with 5 μL of sap. Fifteen such serial passages were performed. Among the nine Ca lineages still present at the end of Phase 1, five were not able to infect *N. tabacum* anymore. Moreover, two lineages lost the ability to produce a systemic infection and only produced local lesions on the inoculated leaf in *N. tabacum*. For these reasons, we decided not to use the Ca lineages in the second evolution phase because they would have been at a lower replication level and the transmission from local lesions to local lesions would have represented evolutionary conditions different from the other lineages and could not have been compared directly. The present experiment was thus started with 60 different lineages and all of them were kept until the end. This evolution experiment, which represents a “common host phase”, will be denominated Phase 2 hereafter.

### Infectivity and virulence measurement

After the 15^th^ passage of Phase 2, infected tissue was collected from each lineage and the viral RNA content was measured by RT-qPCR as described in [24]. The obtained quantification was used to prepare saps of equal viral RNA concentrations. Each of these saps was mechanically inoculated (5 μL of sap on one leaf) on *N. tabacum* and a variable number of other host species depending on the specific evolutionary history of the lineages (Additional file 1: Table S1). This was done, instead of a complete cross design, to limit the measures to cases with relevant biological meaning (inoculation on host present at an anterior step of experimental evolution) and thus reduce the total number of combinations. Plants of all species were at the four leaves stage when inoculated. The replication was of four plants per lineage × host species combination. For practical reasons, the inoculation was spread over four days, with the replicate lineages within an evolutionary history split between the days. Additionally, each day, four plants of each species were inoculated with inoculation buffer, as controls. Before inoculation, the aerial part of each plant was measured (from the basis of the stem to the apex) with a precision of 0.5 cm. At 21 dpi, each plant was checked individually and the presence of symptoms was noted, to then calculate infectivity. The aerial part was measured with a precision of 0.5 cm and weighted with a precision of 10 mg (with a Kern 440-35N balance, Kern and Sohn Gmbh). We define virulence as the degree of damage caused to a plant by the viral infection, which is assumed to be negatively correlated with host fitness [53,54]. We calculated the virulence expressed on size as:

$$V_{S}\left(E_{i}H_{j}\right)=1–\mathrm{\Delta S}\left(E_{i}H_{j}\right)/\mathrm{\Delta S}\left(\text{control}\right)$$

where *V*_*S*_(*E*_*i*_*H*_*j*_) is the virulence expressed on size of the *i*^th^ replicate of evolutionary history *E* when inoculated on the *j*^th^ replicate of host *H* and Δ*S* is the difference in size between the day of infection and 21 dpi. A similar virulence index was obtained from the weight, *V*_*W*_(*E*_*i*_*H*_*j*_). However, Δ*W* cannot be calculated directly because it is impossible to weigh the plant before inoculation. We thus established the correlation between weight and size for each species for plants of the same age as the ones we inoculated on an independent cohort of healthy plants reared in the same conditions as the one used for infectivity and virulence measurements. Using the correlation for each species and the size at inoculation, we could estimate the expected weight at inoculation for each plant and thus estimate Δ*W*.

### Genomic consensus sequence

Total RNA was extracted from infected tissue of the 60 experimentally evolved lineages with the InviTrap® Spin Plant RNA Mini Kit (Invitek) following manufacturer’s instructions. Total RNA concentration was measured spectrophotometrically and all samples were diluted to 50 ng μL^−1^. The TEV genome was amplified in three overlapping fragments and Sanger-sequenced, following the same strategy as in [24]. This method allows obtaining the consensus sequence from nucleotide 48 to nucleotide 9492, *i.e.* 99% of the full genome and 100% of the coding sequence. The average coverage using this sequencing strategy was 2.48. The genomes were assembled and the mutations were identified using the Staden 2.0.0b7 package.

Our estimate of virulence and infectivity were at the population level and we did not explore the variability of these variables within each replicate lineage. For this reason, we directly sequenced PCR amplified virus population cDNAs rather than sequencing multiple clones isolated from the population. Such a consensus sequencing approach allows detecting the dominant nucleotide at each base position. When multiple sequencing reads showed clearly the presence of two peaks at one position, the lineage was considered to be polymorphic at this position. However, it is impossible with this method to measure the frequency of each allele and any mutation that did not reach a frequency detectable on the chromatogram was not recorded. The real within population diversity is thus higher than the one reported here.

## Competing interest

The authors declare that they have no competing interests.

## Authors’ contributions

SB conceived the study, performed the experimental evolution and sequencing, analyzed the data and wrote the manuscript. GL collaborated during experimental evolution. SFE conceived the study, participated in data analysis and helped writing the manuscript. All authors read and approved the final manuscript.

## Supplementary Material

Additional file 1 Tables S1 and S2 and Figure S1. Click here for file
