# Supplementary material for: Genotypic but not phenotypic historical contingency revealed by viral experimental evolution
Source: BMC Evol Biol. 2013 Feb 19;13:46. doi: 10.1186/1471-2148-13-46 (PMC3598485; doi:10.1186/1471-2148-13-46)

# **Supplemental material**

| **Table S1:** Evolutionary history × host species used for the measure of infectivity and virulence of the experimentally evolved lineages. | | | | | | | |
| --- | --- | --- | --- | --- | --- | --- | --- |
| **Evolutionary history** | ***N. tabacum*** | ***N. benthamiana*** | | | ***D. stramonium*** | | ***C. annuum*** |
| *Nb* | X | | X |  | |  | |
| *NbNt* | X | | X |  | |  | |
| *Nt* | X | | X | X | | X | |
| *NtCa* | X | |  |  | | X | |
| *DsCa* | X | |  | X | | X | |
| *Ds* | X | |  | X | | X | |

| **Table S2:**  List of individual mutations that happened between the end of Phase 1 and the end of Phase 2, for each independent lineage. The last column indicates the characteristic of the mutation, taking into account what had happened at the same position during Phase 1. The different types mentioned here correspond to the colour code in Figure 2. “Rev. from poly. state” stands for “reversion from a polymorphic state”. | | | | |
| --- | --- | --- | --- | --- |
| **Lineage** | **Covered** | **Changes from end of Phase 1 to end of Phase 2** | **Protein and amino acid change** | **Type of mutation** |
| *Nb1* | 54 - 9489 | G5055G/A | CI; Syn | New |
| *Nb2* | 54 - 9493 | G4527A | CI; Syn | New |
| *Nb3* | 54 - 9489 | G258G/A | P1; Syn | New |
|  |  | A626G/A | P1; K161R | New |
| *Nb4* | 51 - 9489 | U579C | P1; Syn | New |
|  |  | G1622A | HC-Pro; Syn | New |
|  |  | U7581A | NIb; Syn | New |
| *Nb5* | 48 - 9492 | G4970A | CI; R1609H | New |
| *Nb6* | 53 - 9492 | G3013G/A | P3; E957K | Reverting |
| *Nb7* | 56 - 9489 | G5241A | CI; Syn | New |
| *Nb8* | 54 - 9493 | A2460G | P3; Syn | New |
|  |  | G3013A | P3; E957K | Reversion |
|  |  | U8489C | NIb; I2830T | New |
| *Nb9* | 57 – 9492 |  |  |  |
| *Nb10* | 49 - 9492 | C7299T/C | NIb; Syn | New |
| *NbNt1* | 50 - 9491 | U1599C | HC-Pro; syn | New |
|  |  | C/U4307U | CI; A1388V | Fixed |
|  |  | U6918C | NIa-Pro, Syn | New |
|  |  | A/G8651A | CP; S2836N | Rev. from poly. state |
|  |  | A8575G | CP; K2811E | New |
| *NbNt2* | 48 - 9493 | G441A | P1; Syn | New |
| *NbNt3* | 2925 - 9489 | G7020A/G | NIb; Syn | New |
| *NbNt4* | 48 - 9492 |  |  |  |
| *NbNt5* | 48 - 9492 | A580A/G | P1; T146A | New |
| *NbNt7* | 57 - 9493 |  |  |  |
| *NbNt8* | 72 - 9493 | A1087G/A | HC-Pro; I315V | New |
| *NbNt9* | 72 - 9493 | U444G/U | P1; N100K | New |
|  |  | A1534G/A | HC-Pro; M464V | New |
|  |  | C4020U/C | CI, Syn | New |
| *NbNt10* | 52 - 9493 | A333A/G | P1; Syn | New |
|  |  | G3013G/A | P3; E957K | Reversing |
|  |  | U5955U/C | VPg; Syn | Reversing |
|  |  | G7273G/A | NIb; I2377V | Reversing |
| *Ds1* | 48 - 9489 | C/U1878C | HC-Pro; Syn | Fixed |
|  |  | G/A6010G | VPg; I1956V | Fixed |
| *Ds2* | 49 - 9492 | G/A1376A | HC-Pro; R411K | Rev. from poly. state |
| *Ds3* | 60 - 9492 | C/U554C | P1; V137A | Rev. from poly. state |
|  |  | U1446C | HC-Pro, Syn | New |
|  |  | A1701G | HC-Pro, Syn | New |
|  |  | A1840G | HC-Pro, T566A | New |
|  |  | C/U7044U | NIb; Syn | Rev. from poly. state |
| *Ds4* | 48 - 9492 |  |  |  |
| *Ds5* | 49 - 9492 | G4185A | CI; Syn | New |
|  |  | G/A7422G | NIb; Syn | Rev. from poly. state |
| *Ds6* | 49 - 9493 | C6028G | VPg; P1962A | New |
| *Ds7* | 48 - 9493 | C1462A | HC-Pro; L440M | New |
|  |  | G2916A/G | P3; Syn | New |
|  |  | C9157U/C | CP; H3005Y | New |
| *Ds8* | 68 - 9493 | 120delU | 5’UTR | New |
|  |  | C4978C/U | CI; Syn | New |
| *Ds9* | 53 - 9493 | C/U388U | P1; H82Y | Rev. from poly. state |
|  |  | G/A1639G | HC-Pro; T499A | Rev. from poly. state |
|  |  | U/C3077U | P3; S978L | Rev. from poly. state |
|  |  | A3652G | CI; T1170A | New |
| *Ds10* | 48 - 9493 |  |  |  |
| *DsCa1* | 48 - 9492 |  |  |  |
| *DsCa2* | 54 - 9493 | U/C6774U | NIa-Pro; Syn | Fixed |
| *DsCa3* | 48 - 9493 | A/G1119A | HC-Pro; Syn | Rev. from poly. state |
|  |  | G/U1620G | HC-Pro; Syn | Rev. from poly. state |
| *DsCa4* | 48 - 9493 | G5187A | CI; Syn | New |
| *DsCa5* | 51 - 9493 | C1207A/C | HC-Pro; P355T | New |
|  |  | G8479A/G | NIb; D2779Y | New |
| *DsCa6* | 77 - 9493 | C7935U/C | NIb; Syn | New |
|  |  | G/A8703G | CP; Syn | Fixed |
| *DsCa7* | 48 - 9493 |  |  |  |
| *DsCa8* | 68 - 9493 | G5448A | CI; Syn | New |
| *DsCa9* | 49 - 9493 | G3798G/A | CI; Syn | New |
| *DsCa10* | 48 - 9493 | A3594G/A | 6K1; Syn | New |
| *Nt1* | 49 - 9490 | C/A259A | P1; Q39K | Rev. from poly. state |
|  |  | G1047A | P1; M301I | New |
|  |  | C2022U | HC-Pro; Syn | New |
|  |  | A3414U/A | P3; E1090D | New |
|  |  | G4818A | CI; Syn | New |
|  |  | A5962G | VPg; I1940V | New |
| *Nt2* | 60 - 9493 | G755A | P1; G204D | New |
|  |  | U2353C | P3, Syn | New |
|  |  | G4611A | CI; Syn | Reversed |
|  |  | A7554A/G | NIb; Syn | New |
| *Nt3* | 48 - 9488 | G/A2733G | P3; Syn | Rev. from poly. state |
|  |  | C/U3688U | CI; Syn | Rev. from poly. state |
|  |  | G4615A/G | CI; V1491I | New |
| *Nt4* | 64 - 9493 | G2145A | HC-Pro; Syn | New |
| *Nt5* | 58 - 9493 | C4293A | CI; Syn | New |
|  |  | U4986C | CI; Syn | New |
|  |  | A6945G | NIa-Pro; Syn | New |
|  |  | C8028U | NIb; Syn | New |
|  |  | U8490G | NIb; I2782M | New |
| *Nt6* | 49 - 9493 | G3043A | P3; D967N | New |
|  |  | U4616U/C | CI; V1491A | New |
| *Nt7* | 48 – 9493 | G/A508A | P1; E122K | Rev. from poly. state |
|  |  | G/A2697G | P3; Syn | Rev. from poly. state |
|  |  | U3540U/A | 6K1; Syn | New |
|  |  | U3699C | CI; Syn | New |
| *Nt8* | 49 - 9493 | G3309C | P3; Q1055H | New |
| *Nt9* | 48 - 9488 | A/U654U | P1; R170S | Rev. from poly. state |
|  |  | G776G/A | P1; S211N | New |
|  |  | C2247C/U | HC-Pro; Syn | New |
|  |  | A4131A/G | CI; Syn | New |
|  |  | C9264C/U | CP; Syn | New |
| *Nt10* | 60 - 9480 | A6805G | VPg; K2221E | New |
| *NtCa1* | 49 - 9493 |  |  |  |
| *NtCa2* | 60 - 9489 | G4609G/U | CI; V1489L | New |
| *NtCa3* | 49 - 9493 | U444C | P1; Syn | New |
|  |  | C5340U | CI; Syn | New |
|  |  | C6264U | VPg; Syn | New |
| *NtCa4* | 49 - 9493 | U3732U/C | CI; Syn | New |
| *NtCa5* | 48 -9493 | U444C | P1; Syn | New |
|  |  | G5334A | CI; Syn | Reversed |
|  |  | C5340U | CI; Syn | New |
|  |  | G5409A | CI; Syn | Reversed |
| *NtCa6* | 48 - 9494 | A4239A/G | CI, Syn | New |
|  |  | U/C5512C | CI; Syn | Rev. from poly. state |
|  |  | C5554C/U | 6K2; H1804Y | New |
|  |  | C5779C/A | VPg; L1879I | New |
|  |  | A6805G | NIa-Pro; K2221E | New |
| *NtCa7* | 109 - 9492 | C113U | 5’UTR | New |
| *NtCa8* | 58 - 9492 | A3490A/C | 6K1; I1116L | New |
|  |  | A7206A/G | NIb; Syn | New |
|  |  | G/A8316G | NIb; Syn | Fixed |
| *NtCa9* | 58 - 9488 | G1002A | P1; Syn | New |
| *NtCa10* | 58 - 9448 | U6540C | NIa-Pro; Syn | New |

**Figure S1**. Experimental design. During the “host specialization phase”, viriones were transferred either on a single host (green lineages) or in alternation on two different hosts (orange lineages). The results of phase 1 evolution are exposed in Bedhomme *et al*. (2012). During the “common host phase”, the lineages were all transmitted on the same host. Each phase was constituted of 15 weekly transfers by mechanical inoculation. Ds stands for *D. stramonium*, Ca for *C. annuum*, Nt for *N. tabacum* and Nb for *N. benthamiana*.


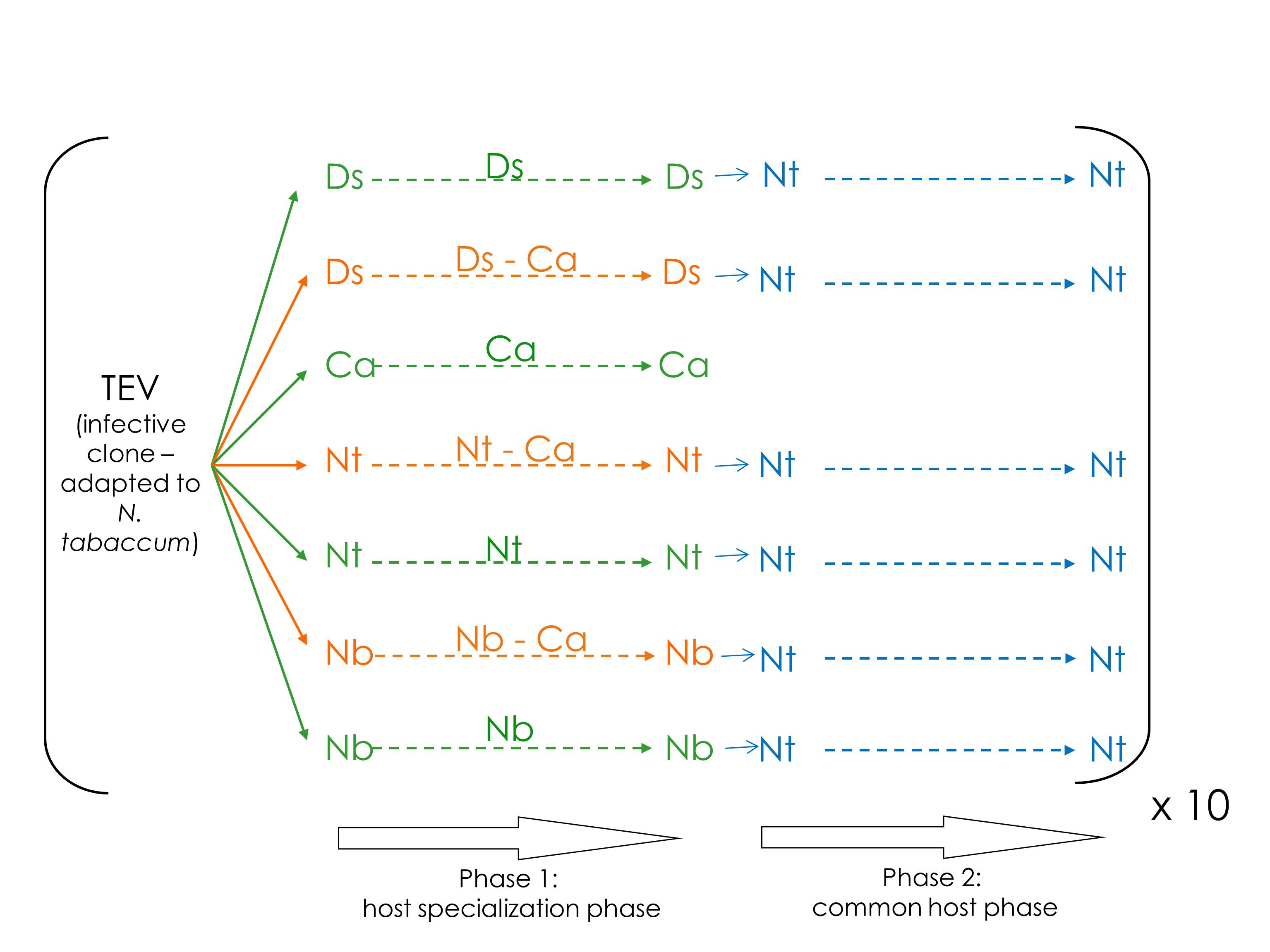

Supplement: Additional file 1 — Tables S1 and S2 and Figure S1. [file 1471-2148-13-46-S1.doc]
